# 2-Amino-5-chloro­pyridinium nitrate

**DOI:** 10.1107/S160053680904149X

**Published:** 2009-10-17

**Authors:** Donia Zaouali Zgolli, Habib Boughzala, Ahmed Driss

**Affiliations:** a Laboratoire de Matériaux et Cristallochimie, Faculté des Sciences, El Manar, 2092 Tunis, Tunisia

## Abstract

The title structure, C_5_H_6_ClN_2_^+^·NO_3_^−^, is held together by extensive hydrogen bonding between the NO_3_^−^ ions and 2-amino-5-chloro­pyridinium H atoms. The cation–anion N—H⋯O hydrogen bonds link the ions into a zigzag- chain which develops parallel to the *b* axis. The structure may be compared with that of the related 2-amino-5-cyano­pyridinium nitrate.

## Related literature

For metal-organic frameworks involving amine derivatives, see: Manzur *et al.* (2007 ▶); Ismayilov *et al.* (2007 ▶); Austria *et al.* (2007 ▶). For related structures, see: Pourayoubi *et al.* (2007 ▶); Rademeyer (2005 ▶, 2007 ▶); Dai (2008 ▶).
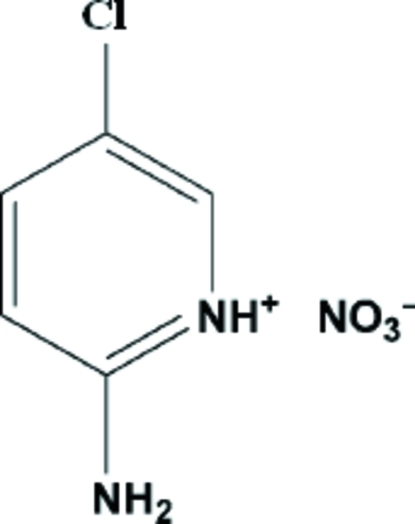


## Experimental

### 

#### Crystal data

C_5_H_6_ClN_2_^+^·NO_3_^−^
*M* *_r_* = 191.58
Monoclinic, 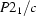

*a* = 4.788 (4) Å
*b* = 13.029 (3) Å
*c* = 12.779 (2) Å
β = 101.445 (18)°
*V* = 781.3 (7) Å^3^
*Z* = 4
Mo *K*α radiation
μ = 0.46 mm^−1^
*T* = 299 K
0.40 × 0.40 × 0.20 mm

#### Data collection

Enraf–Nonius CAD-4 diffractometer
Absorption correction: ψ scan (North *et al.*, 1968 ▶) *T*_min_ = 0.838, *T*_max_ = 0.914
2057 measured reflections
1691 independent reflections
1312 reflections with *I* > 2σ(*I*)
*R*_int_ = 0.017
2 standard reflections frequency: 120 min intensity decay: none

#### Refinement

*R*[*F* ^2^ > 2σ(*F* ^2^)] = 0.037
*wR*(*F* ^2^) = 0.106
*S* = 1.02
1691 reflections
109 parameters
H-atom parameters constrained
Δρ_max_ = 0.19 e Å^−3^
Δρ_min_ = −0.25 e Å^−3^

### 

Data collection: *CAD-4 EXPRESS* (Enraf–Nonius, 1994 ▶); cell refinement: *CAD-4 EXPRESS*; data reduction: *XCAD4* (Harms & Wocadlo, 1995 ▶); program(s) used to solve structure: *SHELXS97* (Sheldrick, 2008 ▶); program(s) used to refine structure: *SHELXL97* (Sheldrick, 2008 ▶); molecular graphics: *ORTEP-3 for Windows* (Farrugia, 1997 ▶) and *PLATON* (Spek, 2009 ▶); software used to prepare material for publication: *WinGX* (Farrugia, 1999 ▶).

## Supplementary Material

Crystal structure: contains datablocks I, global. DOI: 10.1107/S160053680904149X/dn2495sup1.cif

Structure factors: contains datablocks I. DOI: 10.1107/S160053680904149X/dn2495Isup2.hkl

Additional supplementary materials:  crystallographic information; 3D view; checkCIF report

## Figures and Tables

**Table 1:** Hydrogen-bond geometry (Å, °)

*D*—H⋯*A*	*D*—H	H⋯*A*	*D*⋯*A*	*D*—H⋯*A*
N2—H2*A*⋯O3	0.86	2.05	2.900 (2)	169
N2—H2*B*⋯O1^i^	0.86	2.06	2.912 (2)	174
N3—H3⋯O2	0.86	1.94	2.800 (2)	179

Symmetry code: (i) 
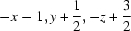
.

**Fig. 1.**
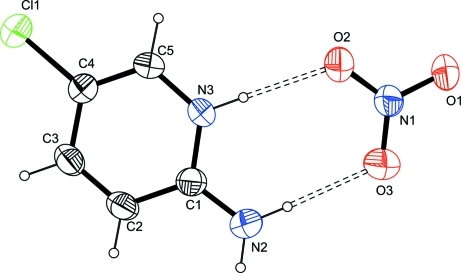
Representation of the asymetric unit of 2-amino-5-chloropyridinium nitrate with the atom labeling scheme. Ellipsoids are drawn at the 30% probability level. H atoms are represented as small spheres of arbitrary radii. Hydrogen bonds are shown as dashed lines.

**Fig. 2.**
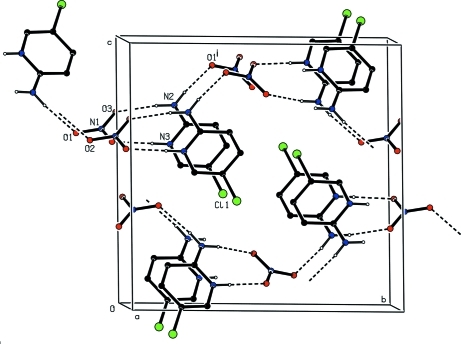
Partial packing view showing the formation of the zigzag chain parallel to the b axis. H atoms not involved in hydrogen bonding have been omitted for clarity.[Symmetry code: (i) -x-1, y+1/2, -z+3/2].

## supplementary crystallographic information

### Comment

Derivatives of the aminoacid are of considerable interest in biological activities and there has been an increased interest in the chemistry of amine derivatives because of the construction of novel metal-organic frameworks (Manzur *et al.*, 2007; Ismayilov *et al.*, 2007; Austria *et al.*, 2007). The crystal structures of 2-amino-5-chloropyridine (Pourayoubi *et al.*, 2007) and their 2-Amino-5-cyanopyridinium nitrate (Dai, 2008) and 2-Aminopyridinium nitrate (Rademeyer, 2007) have been reported in literature. In this paper, we present the X-ray single-crystal structure of 2- Amino-5-chloropyridinium nitrate (I).

The title compound (I) contains an organic cation and a (NO_3_)^-^ anion (Fig1). The cation is roughly planar with the largest deviation from the mean plane being 0.0553 (9)Å. The NO3 anion is slightly twisted with respect to the pyridinium ring making a dihedral angle of 17.2 (1)°

The monoprotonated chloropyridinium cation (C_5_H_6_ClN_2_)^+ ^and the nitrate anion (NO_3_)^- ^are linked by N-H···O which forms a zig-zag like chain parallel to the b axis (Table 1, Fig 2).

The crystal structure of 2-Amino-5-cyanopyridinium nitrate (II) (Dai, 2008) was recently published. The title compound (I) and this related compound (II) are isostructural. In both molecules, the asymmetric unit contains an organic cation 2-Amino-5-Xpyridinium (*X*: chloride (I), C≡N nitrile (II)) and a nitrate anion. They have the same space group (*P*2_1_/*c*) and they are characterized by two-dimensional hydrogen-bonded networks.

The distances and angles of the present 2-amino-5-chloropyridinium nitrate molecule are consistent with the values reported in the literature of 2-aminopyridinium nitrate and 4-aminopyridinium nitrate molecules (Rademeyer 2005;2007).

### Experimental

2-Amino-5-chloropridine was dissolved in the solution of methanol and nitric acid (0.5 ml). Yellow crystals were obtained after a month on slow evaporation.

### Refinement

All H atoms attached to C atoms and N atoms were fixed geometrically and treated as riding with C—H = 0.93Å and N—H = 0.86 Å with U_iso_(H) = 1.2U_eq_(C or N).

### Crystal data

**Table:**

C_5_H_6_ClN_2_^+^·NO_3_^−^	*F*(000) = 392.0
*M**_r_* = 191.58	*D*_x_ = 1.620 Mg m^−^^3^
Monoclinic, *P*2_1_/*c*	Mo *K*α radiation, λ = 0.71073 Å
Hall symbol: -P 2ybc	Cell parameters from 25 reflections
*a* = 4.788 (4) Å	θ = 10–15°
*b* = 13.029 (3) Å	µ = 0.46 mm^−^^1^
*c* = 12.779 (2) Å	*T* = 299 K
β = 101.445 (18)°	Prism, yellow
*V* = 781.3 (7) Å^3^	0.40 × 0.40 × 0.20 mm
*Z* = 4	

### Data collection

**Table:**

Enraf–Nonius CAD-4 diffractometer	1312 reflections with *I* > 2σ(*I*)
Radiation source: fine-focus sealed tube	*R*_int_ = 0.017
graphite	θ_max_ = 27.0°, θ_min_ = 2.3°
Nonprofiled ω/2θ scans	*h* = −1→6
Absorption correction: ψ scan (North *et al.*, 1968)	*k* = −16→1
*T*_min_ = 0.838, *T*_max_ = 0.914	*l* = −16→15
2057 measured reflections	2 standard reflections every 120 min
1691 independent reflections	intensity decay: none

### Refinement

**Table:**

Refinement on *F*^2^	Primary atom site location: structure-invariant direct methods
Least-squares matrix: full	Secondary atom site location: difference Fourier map
*R*[*F*^2^ > 2σ(*F*^2^)] = 0.037	Hydrogen site location: inferred from neighbouring sites
*wR*(*F*^2^) = 0.106	H-atom parameters constrained
*S* = 1.02	*w* = 1/[σ^2^(*F*_o_^2^) + (0.0566*P*)^2^ + 0.1965*P*] where *P* = (*F*_o_^2^ + 2*F*_c_^2^)/3
1691 reflections	(Δ/σ)_max_ < 0.001
109 parameters	Δρ_max_ = 0.19 e Å^−^^3^
0 restraints	Δρ_min_ = −0.25 e Å^−^^3^

### Special details

**Table:**

Experimental. Number of psi-scan sets used was 3 Theta correction was applied. Averaged transmission function was used. No Fourier smoothing was applied (North *et al.*, 1968).
Geometry. All esds (except the esd in the dihedral angle between two l.s. planes) are estimated using the full covariance matrix. The cell esds are taken into account individually in the estimation of esds in distances, angles and torsion angles; correlations between esds in cell parameters are only used when they are defined by crystal symmetry. An approximate (isotropic) treatment of cell esds is used for estimating esds involving l.s. planes.
Refinement. Refinement of *F*^2^ against ALL reflections. The weighted *R*-factor *wR* and goodness of fit *S* are based on *F*^2^, conventional *R*-factors *R* are based on *F*, with *F* set to zero for negative *F*^2^. The threshold expression of *F*^2^ > σ(*F*^2^) is used only for calculating *R*-factors(gt) *etc*. and is not relevant to the choice of reflections for refinement. *R*-factors based on *F*^2^ are statistically about twice as large as those based on *F*, and *R*- factors based on ALL data will be even larger.

### Fractional atomic coordinates and isotropic or equivalent isotropic displacement parameters (Å^2^)

**Table:**

	*x*	*y*	*z*	*U*_iso_*/*U*_eq_	
Cl1	0.35469 (12)	0.37026 (4)	0.42068 (4)	0.0558 (2)	
N1	−0.4403 (4)	−0.03452 (11)	0.63887 (12)	0.0404 (4)	
N2	−0.2955 (4)	0.22776 (13)	0.73365 (13)	0.0484 (4)	
H2A	−0.3487	0.1646	0.7301	0.058*	
H2B	−0.3440	0.2679	0.7804	0.058*	
N3	−0.0639 (3)	0.19881 (11)	0.59367 (12)	0.0373 (3)	
H3	−0.1153	0.1356	0.5943	0.045*	
C1	−0.1377 (4)	0.26344 (13)	0.66666 (13)	0.0349 (4)	
C2	−0.0428 (4)	0.36615 (14)	0.66731 (15)	0.0399 (4)	
H2	−0.0854	0.4122	0.7175	0.048*	
C3	0.1112 (4)	0.39778 (15)	0.59430 (15)	0.0411 (4)	
H3A	0.1751	0.4652	0.5950	0.049*	
C4	0.1737 (4)	0.32811 (14)	0.51762 (14)	0.0373 (4)	
C5	0.0878 (4)	0.22883 (14)	0.51921 (14)	0.0383 (4)	
H5	0.1318	0.1819	0.4701	0.046*	
O1	−0.5381 (3)	−0.12309 (10)	0.62043 (12)	0.0545 (4)	
O2	−0.2376 (3)	−0.00614 (11)	0.59683 (13)	0.0523 (4)	
O3	−0.5384 (4)	0.02366 (12)	0.70018 (14)	0.0635 (4)	

### Atomic displacement parameters (Å^2^)

**Table:**

	*U*^11^	*U*^22^	*U*^33^	*U*^12^	*U*^13^	*U*^23^
Cl1	0.0681 (4)	0.0477 (3)	0.0615 (3)	−0.0101 (2)	0.0366 (3)	−0.0017 (2)
N1	0.0517 (9)	0.0326 (8)	0.0380 (8)	−0.0009 (7)	0.0115 (7)	0.0026 (6)
N2	0.0649 (10)	0.0414 (9)	0.0454 (9)	−0.0019 (8)	0.0269 (8)	−0.0030 (7)
N3	0.0455 (8)	0.0264 (7)	0.0427 (8)	−0.0034 (6)	0.0156 (7)	−0.0047 (6)
C1	0.0387 (9)	0.0339 (8)	0.0324 (8)	0.0019 (7)	0.0081 (7)	−0.0013 (7)
C2	0.0495 (10)	0.0318 (9)	0.0389 (9)	0.0003 (7)	0.0098 (8)	−0.0077 (7)
C3	0.0495 (10)	0.0290 (8)	0.0445 (10)	−0.0039 (7)	0.0088 (8)	−0.0051 (7)
C4	0.0384 (9)	0.0362 (9)	0.0396 (9)	−0.0013 (7)	0.0136 (7)	−0.0006 (7)
C5	0.0444 (9)	0.0333 (9)	0.0406 (9)	−0.0005 (7)	0.0171 (8)	−0.0082 (7)
O1	0.0784 (10)	0.0347 (7)	0.0552 (8)	−0.0152 (7)	0.0251 (8)	−0.0006 (6)
O2	0.0590 (9)	0.0369 (7)	0.0685 (9)	−0.0069 (6)	0.0308 (8)	−0.0010 (6)
O3	0.0788 (11)	0.0513 (9)	0.0708 (10)	−0.0089 (8)	0.0403 (9)	−0.0158 (8)

### Geometric parameters (Å, °)

**Table:**

Cl1—C4	1.7358 (19)	N3—H3	0.8600
N1—O3	1.247 (2)	C1—C2	1.413 (3)
N1—O1	1.250 (2)	C2—C3	1.363 (3)
N1—O2	1.255 (2)	C2—H2	0.9300
N2—C1	1.333 (2)	C3—C4	1.411 (3)
N2—H2A	0.8600	C3—H3A	0.9300
N2—H2B	0.8600	C4—C5	1.359 (3)
N3—C1	1.355 (2)	C5—H5	0.9300
N3—C5	1.364 (2)		
			
O3—N1—O1	120.41 (17)	C3—C2—C1	119.96 (16)
O3—N1—O2	120.64 (16)	C3—C2—H2	120.0
O1—N1—O2	118.93 (16)	C1—C2—H2	120.0
C1—N2—H2A	120.0	C2—C3—C4	119.95 (17)
C1—N2—H2B	120.0	C2—C3—H3A	120.0
H2A—N2—H2B	120.0	C4—C3—H3A	120.0
C1—N3—C5	123.36 (15)	C5—C4—C3	119.70 (17)
C1—N3—H3	118.3	C5—C4—Cl1	120.49 (14)
C5—N3—H3	118.3	C3—C4—Cl1	119.80 (14)
N2—C1—N3	118.95 (16)	C4—C5—N3	119.23 (16)
N2—C1—C2	123.31 (16)	C4—C5—H5	120.4
N3—C1—C2	117.74 (15)	N3—C5—H5	120.4

### Hydrogen-bond geometry (Å, °)

**Table:**

*D*—H···*A*	*D*—H	H···*A*	*D*···*A*	*D*—H···*A*
N2—H2A···O3	0.86	2.05	2.900 (2)	169
N2—H2B···O1^i^	0.86	2.06	2.912 (2)	174
N3—H3···O2	0.86	1.94	2.800 (2)	179

Symmetry codes: (i) −*x*−1, *y*+1/2, −*z*+3/2.
